# The effect of kidney function on guideline‐directed medical therapy implementation and prognosis in heart failure with reduced ejection fraction

**DOI:** 10.1002/clc.24244

**Published:** 2024-02-25

**Authors:** Fanni Bánfi‐Bacsárdi, Dávid Pilecky, Máté Vámos, Zsuzsanna Majoros, Gábor Márton Török, Tünde Dóra Borsányi, Miklós Dékány, Balázs Solymossi, Péter Andréka, Gábor Zoltán Duray, Róbert Gábor Kiss, Noémi Nyolczas, Balázs Muk

**Affiliations:** ^1^ Department of Cardiology Central Hospital of Northern Pest ‐ Military Hospital Budapest Hungary; ^2^ Department of Adult Cardiology Gottsegen National Cardiovascular Center Budapest Hungary; ^3^ Doctoral School of Clinical Medicine University of Szeged Szeged Hungary; ^4^ Cardiac Electrophysiology Division, Cardiology Center, Department of Internal Medicine University of Szeged Szeged Hungary; ^5^ Heart and Vascular Center Semmelweis University Budapest Hungary

**Keywords:** guideline‐directed medical therapy, heart failure with reduced ejection fraction, HFrEF, kidney dysfunction, prognosis

## Abstract

**Background:**

Kidney dysfunction (KD) is a main limiting factor of applying guideline‐directed medical therapy (GDMT) and reaching the recommended target doses (TD) in heart failure (HF) with reduced ejection fraction (HFrEF).

**Hypothesis:**

We aimed to assess the success of optimization, long‐term applicability, and adherence of neurohormonal antagonist triple therapy (TT:RASi [ACEi/ARB/ARNI] + βB + MRA) according to the KD after a HF hospitalization and to investigate its impact on prognosis.

**Methods:**

The data of 247 real‐world, consecutive patients were analyzed who were hospitalized in 2019−2021 for HFrEF and then were followed‐up for 1 year. The application and the ratio of reached TD of TT at hospital discharge and at 1 year were assessed comparing KD categories (eGFR: ≥90, 60−89, 45−59, 30−44, <30 mL/min/1.73 m^2^). Moreover, 1‐year all‐cause mortality and rehospitalization rates in KD subgroups were investigated.

**Results:**

Majority of the patients received TT at hospital discharge (77%) and at 1 year (73%). More severe KD led to a lower application ratio (*p* < .05) of TT (92%, 88%, 80%, 73%, 31%) at discharge and at 1 year (81%, 76%, 76%, 68%, 40%). Patients with more severe KD were less likely (*p* < .05) to receive TD of MRA (81%, 68%, 78%, 61%, 52%) at discharge and a RASi (53%, 49%, 45%, 21%, 27%) at 1 year.

One‐year all‐cause mortality (14%, 15%, 16%, 33%, 48%, *p* < .001), the ratio of all‐cause rehospitalizations (30%, 35%, 40%, 43%, 52%, *p* = .028), and rehospitalizations for HF (8%, 13%, 18%, 20%, 38%, *p* = .001) were significantly higher in more severe KD categories.

**Conclusions:**

KD unfavorably affects the application of TT in HFrEF, however poorer mortality and rehospitalization rates among them highlight the role of the conscious implementation and up‐titration of GDMT.

## Graphical abstract

The implementation and maintenance of guideline‐directed medical therapy (GDMT) were possible in a real‐world population with heart failure with reduced ejection fraction (HFrEF) throughout a broad spectrum of kidney dysfunction (KD). KD unfavorably affected triple therapy application, but poorer prognosis highlights the role of the optimization of GDMT in KD.

ACEI, angiotensin‐converting enzyme inhibitor; ARB, angiotensin receptor blocker; ARNI, angiotensin receptor‐neprilysin inhibitor; βB, beta‐blocker; eGFR, estimated glomerular filtration rate; GDMT, guideline‐directed medical therapy; HF, heart failure; HFrEF, heart failure with reduced ejection fraction; MRA, mineralocorticoid receptor antagonist; RASi, renin‐angiotensin system inhibitor.

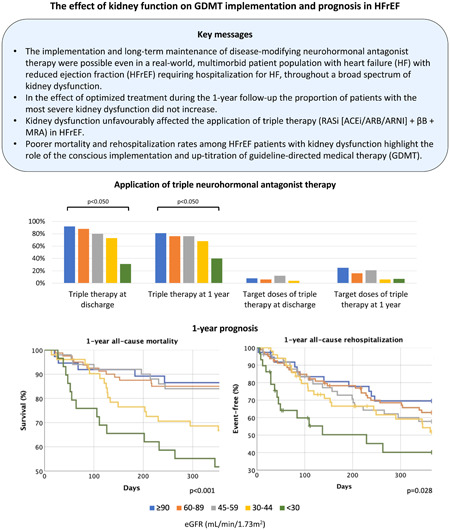

**Abbreviations**
ACEI: angiotensin‐converting enzyme inhibitor
ARB: angiotensin receptor blocker
ARNI: angiotensin receptor‐neprilysin inhibitor
CKD: chronic kidney disease
CKD‐EPI: Chronic Kidney Disease Epidemiology Collaboration
CRT: cardiac resynchronization therapy
CRT‐D: CRT with defibrillator
CRT‐P: CRT without defibrillator
eGFR: estimated glomerular filtration rate
ESC: European Society of Cardiology
FUP: follow‐up
GDMT: guideline‐directed medical therapy
HF: heart failure
HFA: Heart Failure Association
HFOC: Heart Failure Outpatient Clinic
HFrEF: heart failure with reduced ejection fraction
ICD: implantable cardioverter‐defibrillator
KD: kidney dysfunction
MRA: mineralocorticoid receptor antagonist
NT‐proBNP: N‐terminal pro‐B‐type natriuretic peptide
RAASi: renin‐angiotensin‐aldosterone system inhibitor
RASi: renin‐angiotensin system inhibitor
SGLT2i: sodium‐glucose cotransporter 2 inhibitor
TD: target doses
βB: beta‐blocker

## 1 INTRODUCTION

In the treatment of heart failure (HF) with reduced ejection fraction (HFrEF), the use of disease‐modifying pharmacotherapy is of paramount importance. The current 2021 European Society of Cardiology (ESC) guidelines for the treatment of HF recommend the parallel, early introduction and up‐titration of the four pillars of HFrEF, including renin‐angiotensin system inhibitors (RASi; angiotensin‐converting enzyme inhibitors [ACEI], angiotensin receptor‐neprilysin inhibitor [ARNI], angiotensin receptor blockers [ARB] in the case of ACEI and ARNI intolerance), beta‐blockers (βB), mineralocorticoid receptor antagonists (MRA), and sodium‐glucose cotransporter 2 inhibitor (SGLT2i) dapagliflozin/empagliflozin.^1^

The implementation of first‐line pharmacotherapy for HFrEF and achieving the recommended target doses (TD) of renin‐angiotensin‐aldosterone system inhibitors (RAASis) and βBs are limited by the high prevalence of comorbidities, of which kidney disease is of particular relevance.^2^, ^3^, ^4^, ^5^ The prevalence of chronic kidney disease (CKD) is estimated at 14% based on the United States Renal Data System 2022 Annual Data Report.^6^ HF and CKD have bidirectional interaction,^7^ sharing common risk factors, pathophysiology, and therapeutics (i.e., RASi), affecting unfavorably each other's prognosis,^8^ CKD may be present in 32%−49% of HF cases, with a greater prevalence in acute HF than in the chronic patient population (59% vs. 42%).^9^ Coexisting CKD is an independent predictor of mortality and hospitalization in HF patients.^7^, ^9^, ^10^

In a large proportion of landmark randomized controlled trials (RCTs) that assessed different therapeutic options in HFrEF, severely impaired renal function and advanced kidney disease were exclusion criteria,^11^, ^12^, ^13^, ^14^, ^15^, ^16^, ^17^, ^18^ so there is a lack of evidence on the safety and efficacy of strategic agents in patient groups with estimated glomerular filtration rate (eGFR) < 30 mL/min/1.73 m^2^. Thus, the penetration of guideline‐directed medical therapy (GDMT) in HFrEF with concomitant CKD is often low.^19^, ^20^, ^21^ Furthermore, it is a shared concern in everyday practice that the novel recommended parallel implementation of quadruple therapy may amplify the renal side‐effect of all forms of medication, increasing the risk of progressive decline in eGFR even in patients with normal renal function.^22^ Accordingly, in parallel with the ESC 2021 HF guidelines, the ESC Heart Failure Association published a consensus document focusing on tailored therapy in HFrEF.^23^ Moreover, there is growing evidence that the maintenance of RASi treatment among patients with more advanced CKD could favorably modify prognosis even with or without HF.^24^

Hence, there is a great need in everyday practice to evaluate the long‐term success of the implementation and optimization of GDMT in HFrEF in relation to the whole spectrum of kidney dysfunction (KD).

In the current study, we aimed to investigate among a consecutive, real‐world patient cohort hospitalized for HFrEF, the application ratio of neurohormonal antagonist therapy, and the proportion of patients at TD of neurohormonal antagonist therapy according to the KD, as well as the 1‐year prognosis and therapy adherence.

## 2 MATERIALS AND METHODS

### 2.1 Study population and design

A retrospective analysis of a consecutive group of real‐world patients with HFrEF hospitalized for signs and symptoms of HF between January 1, 2019 and October 31, 2021 at the HF Unit of the Department of Cardiology, Medical Centre, Hungarian Defence Forces, tertiary cardiology center was performed.

Intrahospital mortality was an exclusion criterion. In the case of multiple hospitalizations of individual patients during the data collection period, the first event was considered in the analysis to avoid redundancy. The follow‐up (FUP) period was 1 year for all patients.

Our retrospective observational study protocol was reviewed and approved by the Institutional Research Ethics Committee of the Hungarian Defence Forces Medical Centre (approval number: KK00/144‐1/2022), and the investigation conforms with the principles outlined in the Declaration of Helsinki.^25^ For our retrospective observational study, no written informed consent was required as our research did not influence the professional medical care of patients, required no intervention, and involved only retrospective data collection in anonymized form.

Patients were classified into five groups to represent the severity of KD, established using hospital discharge eGFR parameters: eGFR ≥90 mL/min/1.73 m^2^, eGFR = 60−89 mL/min/1.73 m^2^, eGFR = 45−59 mL/min/1.73 m^2^, eGFR = 30−44 mL/min/1.73 m^2^, eGFR < 30 mL/min/1.73 m^2^ groups were formed in concordance with the KDIGO (Kidney Disease: Improving Global Outcomes) classification.^26^ The Chronic Kidney Disease Epidemiology Collaboration (CKD‐EPI) equation was used to calculate eGFR values as a sensitivity analysis. The proportion of patients with significant eGFR decrease occurring within index hospitalization (defined as >15% decline in eGFR value between admission and discharge^27^ was also evaluated.

Our study aimed to assess the following issues among a consecutive, real‐world patient cohort hospitalized for HFrEF:

1. The success of the implementation of complex neurohormonal antagonist therapy (RASi: ACEI/ARB/ARNI, βB, MRA) according to the severity of KD at hospital discharge and at 1 year,
2. The proportion of patients receiving the TD of neurohormonal antagonist therapy according to the severity of KD at hospital discharge and at 1 year,
3. Therapy adherence during 1‐year FUP period,
4. The 1‐year prognosis (all‐cause mortality, all‐cause hospitalization, cardiovascular rehospitalization, and rehospitalization for HF) across the whole spectrum of KD, and
5. The independent predictors of the application of the triple therapy (RASi: ACEI/ARB/ARNI + βB + MRA) at discharge and of the 1‐year all‐cause mortality.

### 2.2 Statistical analysis

The clinical data for the study were obtained from our hospital's electronic system, and mortality data were obtained through the electronic social insurance number validity documentation interface of the National Health Insurance Fund of Hungary. Data were recorded in anonymized form in a Microsoft Excel spreadsheet (Microsoft Corporation), and statistical analysis was undertaken using IBM SPSS Statistics 26.0.

The distribution of continuous variables was tested with the Shapiro−Wilk normality test. Based on non‐Gaussian distribution, continuous variables are presented as a median and interquartile range, while categorical variables are presented as absolute numbers and percentages. The main characteristics of the five KD categories were compared with *χ* ^2^ test for categoric variables and with Kruskal−Wallis test for continues variables.

The implementation rate and the proportion of patients at TD of neurohormonal antagonist therapy were compared with the Pearson's *χ* ^2^ test among the five KD categories at hospital discharge and at 1 year. At three different time points (at hospital admission, discharge, and 1 year) for each KD subgroup, eGFR values were compared using the Friedman test. The subgroups of patients with eGFR decline >15% versus eGFR decline ≤15% were compared with a Fisher test.

Mortality and rehospitalization rates were assessed using Kaplan−Meier analysis and log‐rank tests. The independent predictors of triple therapy application at discharge were investigated with univariate and multivariate logistic regression, while the independent predictors of 1‐year all‐cause mortality were analyzed with univariate and multivariate Cox regression. Statistical significance was defined as *p* < .050.

## 3 RESULTS

### 3.1 Patient population

Data from a cohort of 247 patients (75% male, median age 66 [56−74] years) were analyzed. Forty‐six percent of the patients were found to have HFrEF—at least partly—due to ischemic etiology. In 32% of patients, the diagnosis of HFrEF was confirmed at hospital admission (de novo HFrEF), while 68% had a preexisting diagnosis of HFrEF before hospital admission. Forty percent of patients required prior hospitalization for HF. Every patient was offered multidisciplinary care at our HF Outpatient Clinic (HFOC) at discharge, however, only 45% of them accepted it for different reasons. The main characteristics of the cohort are presented in Table 1.

**Table 1:** Main characteristics of the study population.

Parameters		*n* = 247 patients
Male gender, *n* (%)		185 (75)
Age, median [IQR], years		66 [56–74]
Duration of hospitalization, median [IQR], days		19 [12–27]
Previous hospitalization primarily due to heart failure, *n* (%)		98 (40)
Follow‐up at our HFOC, *n* (%)		110 (45)
Time of HFrEF diagnosis	de novo HFrEF, *n* (%)	79 (32)
	Previously diagnosed HFrEF, *n* (%)	168 (68)
Heart failure etiology	Ischemic, *n* (%)	114 (46)
	Nonischemic/unknown, *n* (%)	133 (54)
LVEF at admission, median [IQR], %		25 [20–30]
Heart rate at admission, median [IQR], min^−1^		88 [74–100]
Systolic blood pressure at admission, median [IQR], mmHg		118 [102–134]
In‐hospital application of positive inotrope and/or inodilator therapy, *n* (%)		4 (2)
Comorbidities		
Diabetes, *n* (%)		100 (40)
Hypertension, *n* (%)		156 (63)
Atrial fibrillation/flutter, *n* (%)		114 (46)
Prehospital diagnosed chronic kidney disease, *n* (%)		50 (20)
Dialysis, *n* (%)		3 (1)
Laboratory parameters at discharge		
Creatinine, median [IQR], μmol/L		111 [87–145]
eGFR, median [IQR], mL/min/1.73 m^2^		59 [39–75]
Potassium, median [IQR], mmol/L		4.36 [4.01–4.68]
Potassium >4.5 mmol/L, *n* (%)		110 (45)
NT‐proBNP, median [IQR], pg/mL		6531 [3350–11 994]
Kidney dysfunction categories established on discharge eGFR parameters		
eGFR ≥ 90 mL/min/1.73 m^2^, *n* (%)		37 (15)
eGFR = 60−89 mL/min/1.73 m^2^, *n* (%)		80 (32)
eGFR = 45−59 mL/min/1.73 m^2^, *n* (%)		50 (20)
eGFR = 30−44 mL/min/1.73 m^2^, *n* (%)		51 (21)
eGFR < 30 mL/min/1.73 m^2^, *n* (%)		29 (12)

Abbreviations: eGFR, estimated glomerular filtration rate; HFOC, Heart Failure Outpatient Clinic; HFrEF, heart failure with reduced ejection fraction; IQR, interquartile range; LVEF, left ventricular ejection fraction; NT‐proBNP, N‐terminal pro‐B‐type natriuretic peptide.

The median left ventricular ejection fraction was 25 [20−30] %, and the N‐terminal pro‐B‐type natriuretic peptide (NT‐proBNP) at admission was 6531 [3350−11 994] pg/mL. The patient population was characterized by frequent comorbidities (diabetes: 40%, hypertension: 63%, atrial fibrillation/flutter: 46%, prehospital CKD: 20%, regular dialysis: 1%). Based on eGFR values measured at discharge, 53% of the patients had eGFR <60 mL/min/1.73 m^2^. The proportions of patients included in the KD groups were as follows: ≥90 mL/min/1.73m^2^: 15%, 60−89 mL/min/1.73 m^2^: 32%, 45−59 mL/min/1.73 m^2^: 20%, 30−44 mL/min/1.73 m^2^: 21%, <30 mL/min/1.73 m^2^: 12%.

At hospital admission, 66% of the patients were on RASi (at TD of RASi: 21%), 68% on βB (at TD of βB: 25%), 58% on MRA therapy (at TD of MRA: 24%), while 42% of them were receiving triple therapy (at TD of triple therapy: 6%). Fifteen percent had an implantable cardioverter‐defibrillator (ICD) without cardiac resynchronization therapy (CRT) and 11% had a CRT with/without defibrillator already implanted (CRT‐P/CRT‐D) at admission (Supporting Information S1: Table 1).

As for the basic characteristics within the five KD categories, significant differences were seen in the age, in the occurrence of comorbidities and in the ratio of de novo HFrEF patients (Supporting Information S1: Table 2).

### 3.2 Changes in renal function during FUP

**Figure 1:**
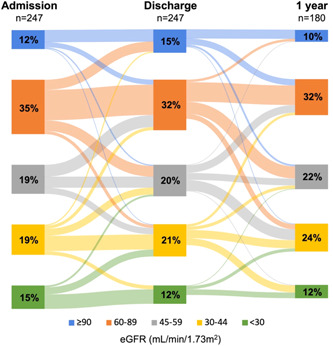
Changes in kidney dysfunction categories during 1‐year follow‐up. eGFR, estimated glomerular filtration rate.

At 1 year, 191 patients were alive whose pharmacotherapy could be analyzed, while 180 patients had eGFR parameters measured at 1 year. Even though median eGFR slightly but significantly worsened among the whole cohort during 1‐year FUP (eGFR: 61 [41−77] vs. 60 [43−79] vs. 55 [38−66] mL/min/1.73 m^2^, *p *< .001; admission vs. discharge vs. 1 year; based on data of 180 patients having eGFR measured at all three time points), the proportion of patients with the most severe KD categories did not increase. Figure 1 shows the distribution of eGFR‐based KD categories.

### 3.3 Application rates of complex neurohormonal antagonist therapy and proportion of patients at TD of triple therapy

At hospital discharge, 77% of the patients received combined triple neurohormonal antagonist therapy (RASi + βB + MRA); 89% received RASi therapy (ACEI/ARB: 72%, ARNI: 17%), 85% had βB, and 95% had MRA medication. The implementation rate of SGLT2i was 10%. At 1 year, of the 191 patients still alive, 73% were on triple therapy; 85% received RASi: 85% (ACEI/ARB: 62%, ARNI: 23%), 89% had βB, and 83% had MRA therapy (Supporting Information S1: Table 1).

**Figure 2:**
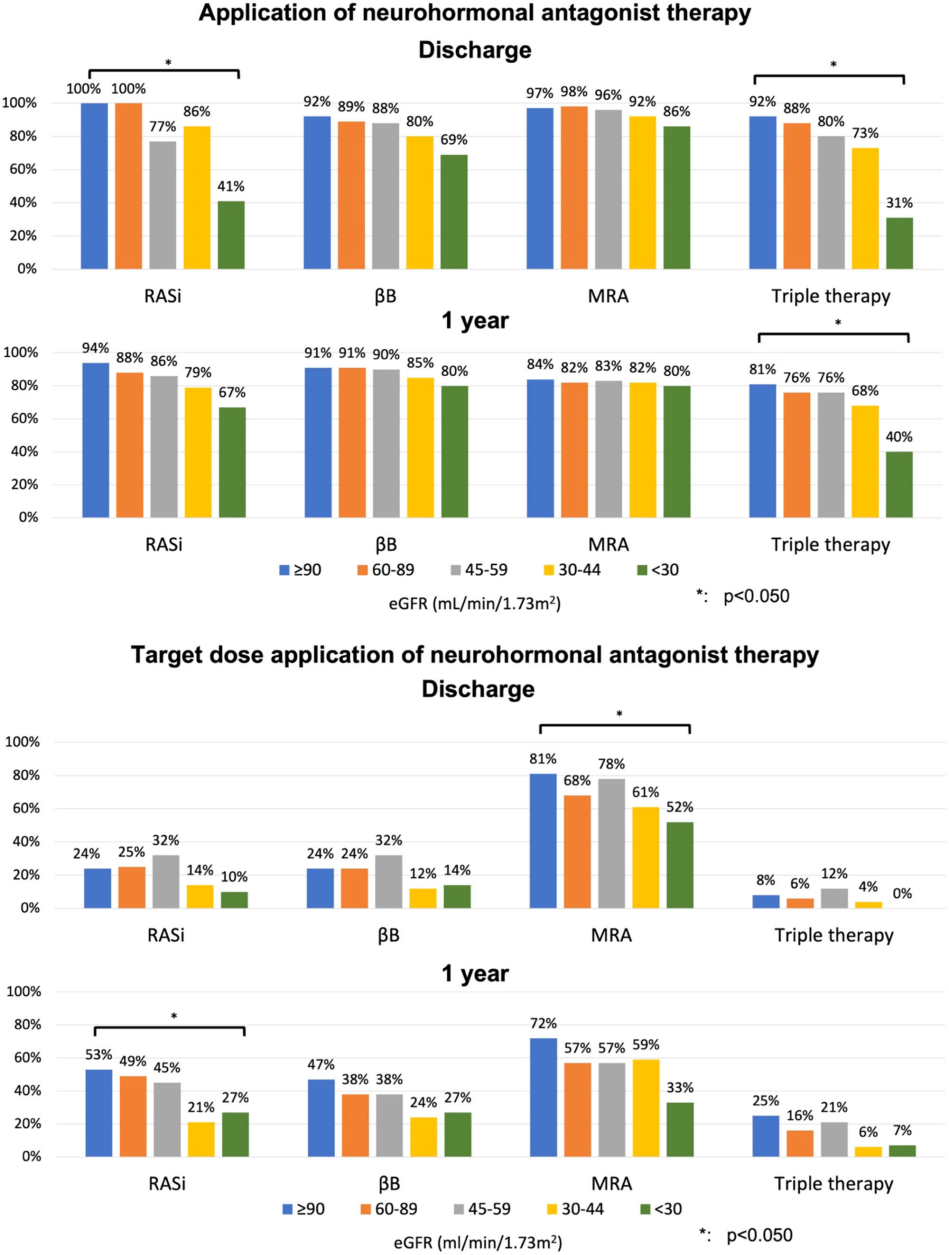
Application rates and target dose application rates of neurohormonal antagonist therapy at discharge and at 1 year. βB, beta‐blocker; eGFR, estimated glomerular filtration rate; MRA, mineralocorticoid receptor antagonist; RASi, renin‐angiotensin system inhibitor.

At hospital discharge, the presence of more severe KD led to a significantly lower application rate of triple therapy (92%, 88%, 80%, 73%, 31%, *p* < .001; in eGFR ≥90, 60−89, 45−59, 30−44, <30 mL/min/1.73 m^2^ groups, respectively) (Figure 2). Among the KD subgroups, there was a significant difference in terms of the proportion of patients on RAS inhibitors, while the ratio of patients on MRA therapy did not differ statistically (Figure 2). Regarding βB application, an unfavorable trend was observed in the more severe KD subgroups (Figure 2). At 1 year, significant deviation persisted among the KD groups regarding the application ratio of triple therapy (81%, 76%, 76%, 68%, 40%, *p* = .033), while no differences were seen in the use of RASi, βB, or MRA medication (Figure 2).

Regarding the TD achieved at discharge, the presence of less favorable renal function did not modify significantly the proportion of patients at TD of RASi, βB, and triple therapy (Figure 2). However, more advanced KD was accompanied by a lower implementation rate of MRA therapy with TD. At 1 year of FUP, TD of a RASi could have been achieved more frequently among patients with more favorable kidney function, while no significant differences were seen regarding the other assessed first‐line HFrEF pharmacotherapies (βB, MRA), and triple therapy (Figure 2).

### 3.4 Therapy adherence

**Figure 3:**
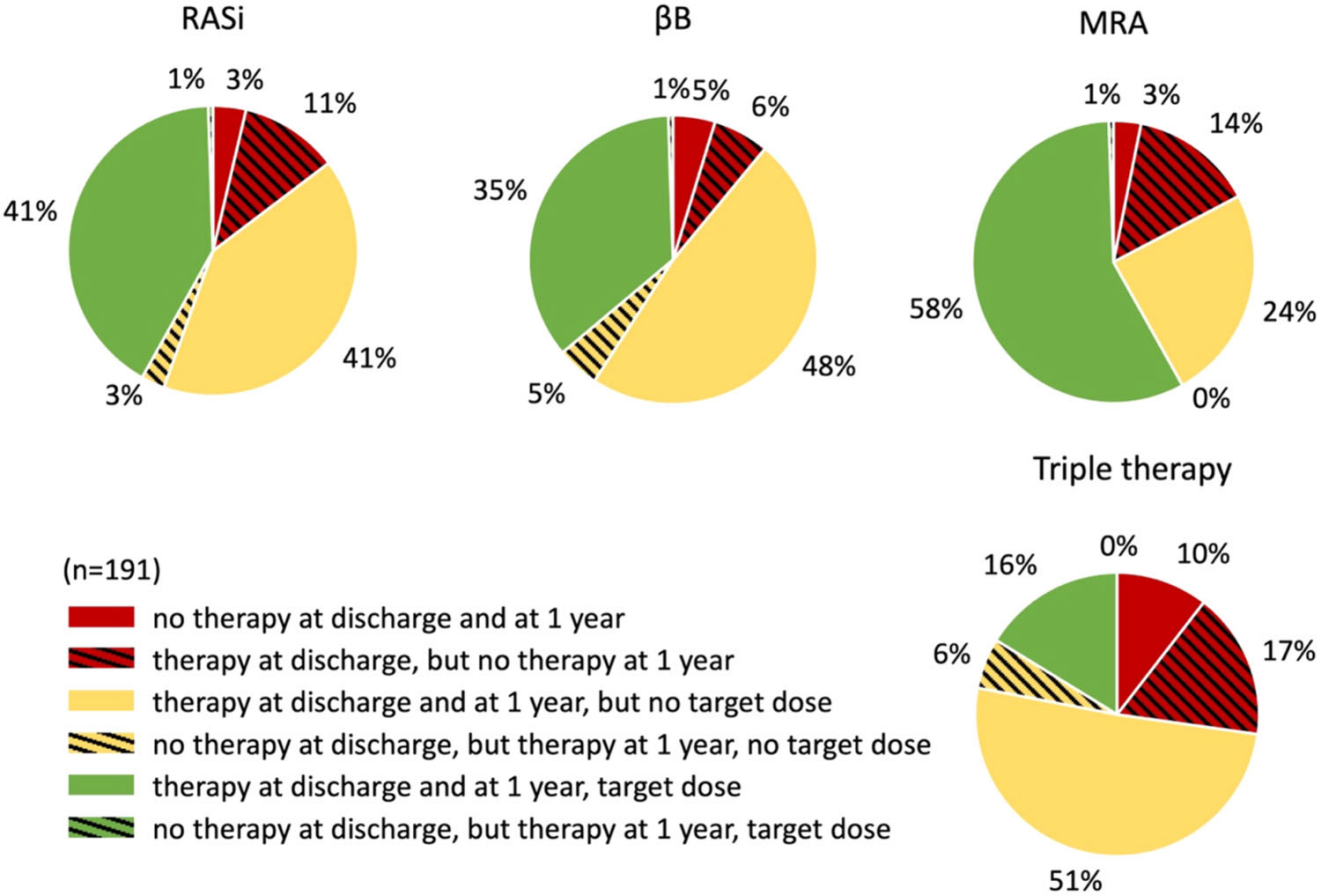
Therapy adherence at 1 year. βB, beta‐blocker; MRA, mineralocorticoid receptor antagonist; RASi, renin‐angiotensin system inhibitor.

The level of therapy adherence was remarkably high in the whole cohort at 1 year (Figure 3). Neurohormonal antagonist drug regime was discontinued only in 6%−17% of patients at 1 year (RASi: 11%, βB: 6%, MRA: 14%, triple therapy: 17%). Moreover, it has to be highlighted that the morbidity and mortality‐reducing therapy was only newly introduced in 1%−6% of the total cohort during the 1‐year FUP after the index hospitalization, and only in an even smaller proportion of the cohort (0%−1%) could therapy have been introduced and titrated to the target dose recommended in the current guidelines.

### 3.5 Prognosis of patients with HFrEF according to KD

**Figure 4:**
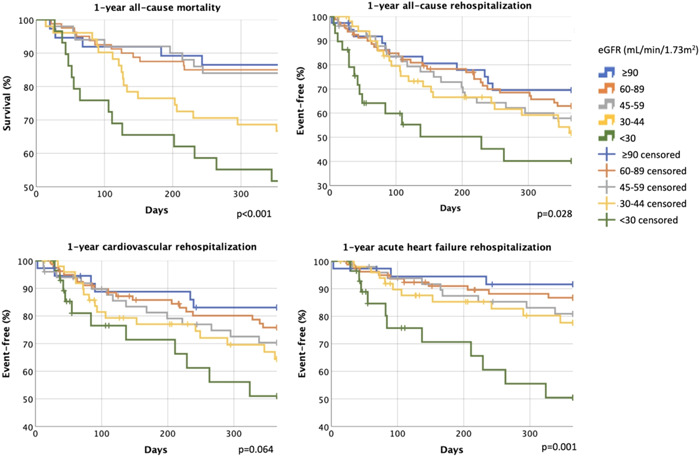
One‐year prognosis (all‐cause mortality, all‐cause rehospitalization, cardiovascular rehospitalization, rehospitalization for acute HF) according to kidney dysfunction categories. eGFR, estimated glomerular filtration rate; HF, heart failure.

One‐year all‐cause mortality of the total cohort was 23%. In the presence of more severe KD, the all‐cause mortality significantly increased, confirmed by the results of Kaplan−Meier analysis and log‐rank test (14%, 15%, 16%, 33%, 48%; *p* < .001; eGFR ≥90, 60−89, 45−59, 30−44, <30 mL/min/1.73 m^2^ groups) (Figure 4).

In the whole cohort, the 1‐year all‐cause rehospitalization rate was 39%, cardiovascular rehospitalization occurred in 26%, while rehospitalization for acute HF in 17%. The 1‐year all‐cause rehospitalization (30%, 35%, 40%, 43%, 52%, *p* = .028) and the rate of rehospitalization for HF (8%, 13%, 18%, 20%, 38%, *p* = .001) were greater among patients with more advanced KD. No significant differences were observed in terms of cardiovascular rehospitalization rates (16%, 23%, 28%, 31%, 38%, *p* = .064) across the KD spectrum, even though an unfavorable trend was detected (Figure 4).

The proportion of patients experiencing a significant decrease in eGFR during index hospitalization was 18%. Among them, the proportion of patients on triple therapy (70% vs. 78%, *p* = .261; eGFR decline >15% vs. ≤15%) and those at TD of triple therapy (9% vs. 6%, *p* = .437; eGFR decline >15% vs. ≤15%) did not differ significantly those for whom the eGFR decline did not occur during index hospitalization.

### 3.6 Independent predictors of the implementation of triple therapy at discharge and of the 1‐year all‐cause mortality

Regarding the predictors of triple therapy implementation at discharge, the well‐known parameters potentially affecting the application of the complex treatment were included in the univariate logistic regression model (Supporting Information S1: Table 3A). On multivariate logistic regression analysis younger age, lower NT‐proBNP level, and absence of diabetes mellitus proved to be the independent predictors of discharge TT application.

Regarding 1‐year all‐cause mortality, younger age, higher systolic blood pressure, FUP at HFOC, and triple therapy at discharge were the independent parameters that reduced 1‐year mortality (Supporting Information S1: Table 3B).

## 4 DISCUSSION

### 4.1 Main findings

Our analysis indicated that the successful implementation of disease‐modifying neurohormonal antagonist therapy is possible even in a real‐world, multimorbid HFrEF patient population requiring hospitalization for HF. Therapy adherence was also remarkably high at 1 year. Our results highlight the importance of in‐hospital therapy optimization, as after index hospitalization the morbidity and mortality‐reducing pillar therapy of HFrEF was initiated only among the minority of the patient cohort.

More severe KD led to a lower application rate of triple therapy at hospital discharge and at 1 year; moreover, patients with more severe KD were less likely to receive TD of MRA at hospital discharge and a RAS inhibitor at 1 year.

All‐cause mortality, all‐cause rehospitalization, and rehospitalization for HF were more frequent among patients with more advanced KD, which may also indicate the importance of early introduction and up‐titration of GDMT of HFrEF.

Younger age, lower NT‐proBNP level and absence of diabetes mellitus facilitated the in‐hospital implementation of triple therapy. One‐year mortality was independently reduced by younger age, higher systolic blood pressure, FUP at HFOC, and triple therapy at hospital discharge.

### 4.2 The relevance of KD and its severity among HFrEF patients

In the evaluated patient population, the prevalence of known CKD (20%) before hospital admission approached that described in the ESC Heart Failure Long‐Term Registry (25.3%)^28^ and the Hungarian Heart Failure Registry (20.3%).^29^ The difference between the proportion of patients with already known CKD and that of patients with de novo diagnosed KD suggests the need for greater awareness for early diagnosis and effective treatment of the relevant, prognosis‐modifying comorbidities in everyday practice.

Although a moderate decline in median eGFR was observed in the whole cohort during 1‐year FUP, the proportion of patients with significant KD—which may be a primary obstacle to therapy optimization—did not increase.^10^

Worsening renal function frequently occurs during acute HF events (as a potential consequence of altered hemodynamic state, neurohormonal changes, and applied pharmacotherapy).^22^, ^30^ Similarly to the analysis of the SOLVD and the PARADIGM‐HF trial^31^, ^32^ our results also confirm that a modest decline in eGFR did not affect the success of the application of triple therapy, which may indicate that even with a moderate worsening of renal function, neurohormonal antagonist treatment can be introduced, optimized safely and effectively, and maintained successfully in a significant proportion of patients.^30^ Moreover, this phenomenon could be used to select those patients who may profit from the life‐saving therapy the most.^33^, ^34^ Furthermore, ARNI and SGLT2i treatment can even reduce the extent of the temporal decline in eGFR,^15^, ^35^ while finerenone also decreases the progression of end‐stage renal disease,^36^ reflecting the nephroprotective effect of these agents.

### 4.3 Application rates of complex neurohormonal antagonist therapy and proportion of patients at TD of triple therapy

In our total multimorbid cohort, the exceptionally high implementation rate of triple therapy (77%) exceeded that of the recently published randomized clinical trials (VICTORIA^37^ 60.7%, GALACTIC‐HF^38^ 66.1%) and of the recently published registries (VICTORIA Registry^39^ 28.4%, CHAMP‐HF Registry^3^ 22.1%) that examined a largely similar patient population with HFrEF. In the GTWG‐HF Registry, even in patients with eGFR ≥90 mL/min/1.73 m^2^, triple therapy application was less than 40%.^2^ In addition, the proportion of patients at the TD of triple therapy was greater in our patient cohort (6%) than in the VICTORIA Registry (1%).^39^ According to a recent publication of Swat et al., among HFrEF patients hospitalized for HF the evidence‐based HF medication was not optimized in 42.8% of the cases or was even reduced in 4.9%, and concomitant renal insufficiency was revealed as an important limiting factor of initiating a new evidence‐based HF medication.^40^

Nonetheless, comparison with different registries (i.e., GWTG‐HF Registry,^2^ SwedeHF,^41^ CARDIOREN registry^42^) is difficult due to the difference between the examined cohorts and study structures (Supporting Information S1: Table 4). In general, all of these registries conclude that evidence‐based HF therapies are less likely to be applied in HF patients with concomitant CKD.^2^, ^41^, ^42^ The GWTG‐HF Registry^2^ database of a cohort of hospitalized patients with symptoms of acute HF may serve as a basis for comparison with our observations. The application rate of RASi, MRA, and triple therapy treatment exceeded that of the GTWG‐HF Registry for all KD groups, while βB medication was similarly distributed in both studies (Supporting Information S1: Table 4). It should be highlighted that the GWTG‐HF Registry did not involve a consecutive patient population, eGFR values were unavailable for all patients, and redundancy caused by multiple hospitalization of the same patients was not eliminated.^43^ Moreover, the analysis of Patel et al. of the GWTG‐HF Registry is limited by the single time point measurement of eGFR.^2^ In contrast, the strength of our study is the multiple time point measurement of renal function parameters and the avoidance of redundancy.

Although patients with CKD stages G4‐G5 were underrepresented in the landmark neurohormonal antagonist studies, a growing number of analyses are assessing the applicability of neurohormonal antagonist therapy in this patient population. The results of the STOP‐ACEI study suggest that discontinuation of RASi only for KD (eGFR <30 mL/min/1.73 m^2^) does not prevent further progression of KD.^44^ However, more data is needed concerning HF patient populations.

HF management in the presence of KD needs a personalized treatment strategy,^30^, ^45^ in which a multidisciplinary approach plays a crucial role.^46^ KD should not preclude the application of HFrEF disease‐modifying medication. However, patients should be closely followed‐up for worsening renal function and potassium levels, and up‐titration may be slower.^47^

### 4.4 Therapy adherence

One‐year therapy adherence was favorable in our patient cohort, exceeding the results of the EVOLUTION‐HF study, which documented the discontinuation of neurohormonal antagonist therapy in 25%−42% of the patients at 1 year.^48^

Our results highlight the fact that among patients not receiving neurohormonal antagonist therapy at hospital discharge, only a minority of cases were introduced during the FUP period. Although hospitalization indicates a poor prognosis for HF, we should take advantage of the opportunities offered by hospitalization to optimize therapy.^49^ The cause of suboptimal therapy adherence and the clinical inertia are multifactorial.^50^ This clearly demonstrates the importance of multidisciplinary HF outpatient care, which can be a key element of the successful FUP of HF patients.^51^

### 4.5 Prognosis of patients with HFrEF according to KD and the main influencing factors of triple therapy application and all‐cause mortality

The 1‐year all‐cause mortality in the whole cohort was comparable to that identified in the RCT examinations of similar patient populations (GALACTIC‐HF^38^ 25.9% in median 21.8‐month FUP; VICTORIA^37^ 21.2% in median 10.8‐month FUP), despite the differences between the examined cohorts. In the acute HF cohort of the ESC Heart Failure Long‐Term Registry, the 1‐year mortality rate (23.6%) was similar to our study (23%), as well as the ratio of 1‐year all‐cause rehospitalizations (18.7% vs. 17%),^28^ even though our patient population was remarkably older, possessed significantly lower left ventricular ejection fraction. Moreover, the proportion of patients with significant KD was higher^28^ in our analysis. These factors may independently be associated with less favorable mortality rates. Our analysis also confirms that a worse prognosis can be expected in the presence of more advanced KD among HFrEF patients.^46^

Our research shows that in‐hospital implementation of conventional triple therapy was independently facilitated by younger age, lower NT‐proBNP level, and absence of diabetes mellitus. The CHAMP‐HF Registry,^3^ BIOSTAT‐CHF registry,^4^ and Victoria Registry^39^ revealed KD as a limiting factor of neurohormonal antagonist therapy initiation and up‐titration. However, eGFR did not prove to be an influencing factor in our multivariate logistic regression analysis.

In our patient cohort, using multivariate Cox regression analysis 1‐year mortality was reduced by younger age, higher systolic blood pressure, FUP at HFOC, and triple therapy at hospital discharge. According to the subanalysis of the ESC Heart Failure Long‐Term Registry,^52^ older age, NYHA class, CKD, lower blood pressure, and higher heart rate were independently associated with higher 1‐year mortality in chronic HFrEF patients. As for acute HF patients, in the recent publication of Lorlowhakarn et al., age, cardiovascular accidents, and NT‐proBNP level were associated with higher 1‐year mortality after acute HF hospitalization.^53^ In the REPORT‐HF registry, patients hospitalized for acute decompensated HF and patients with worsening renal function had the highest mortality rates.^54^ In accordance with our findings, in the international cohort study by Lam et al., renal function was not revealed as an independent predictor of mortality. However, age and systolic blood pressure had a significant effect.^55^

## 5 CONCLUSIONS

KD is one of the main limiting factors of GDMT application and causes of clinical inertia among HFrEF patients. Our results confirm that the implementation and up‐titration of GDMT are possible in everyday clinical practice, even in a multimorbid HFrEF patient population requiring hospitalization for HF, with high therapy adherence during the FUP period. Moreover, triple therapy can be introduced and optimized safely in a large proportion of patients with significant KD. However, renal impairment clearly has a negative impact on the implementation of life‐saving first‐line HFrEF therapy. Mortality and rehospitalization rates among patients with HFrEF with KD are unfavorable, which may indicate the importance of introducing the disease‐modifying drug therapy of HFrEF, as recommended in the current HF guidelines.

### 5.1 Strengths and limitations

The strength of our observational study is that it examines a real‐world, consecutive, unselected patient population, and provides data on the implementation of guideline recommendations in everyday clinical practice. Redundancy in patient inclusion was avoided, and eGFR parameters were based on multiple measurements. One‐year FUP was completed in all patients, providing data on long‐term kidney function parameters, medications, therapy adherence and prognosis, which is rare in the literature to our knowledge and may be of niche importance.

The patient population of our single‐center study were exclusively Caucasian, so our results and conclusions cannot be applied with certainty outside this group. The rate of ARNI use may have been influenced by the reimbursement regulations in Hungary. As the results of the SGLT2i landmark trials (DAPA‐HF,^16^ EMPEROR‐REDUCED^17^) were published during our study and were only incorporated into the 2021 ESC HF guidelines [11], the use rates of dapagliflozin and empagliflozin were not assessed in the present study. Other confounding factors modifying triple therapy implementation and mortality cannot be excluded. The measurement of albuminuria was not an obligatory pillar of the diagnostic pathway associated with our daily routine; hence, it was not used in our analysis.

## CONFLICT OF INTEREST STATEMENT

The authors declare no conflict of interest.

## Supporting information

Supporting information.

## Data Availability

The data that support the findings of this study are available from the corresponding author upon reasonable request. All data generated or analyzed during this study are included in this article. Further enquiries can be directed to the corresponding author.
