# Supplementary material for: The effect of kidney function on guideline‐directed medical therapy implementation and prognosis in heart failure with reduced ejection fraction
Source: Clin Cardiol. 2024 Feb 25;47(2):e24244. doi: 10.1002/clc.24244 (PMC10894619; doi:10.1002/clc.24244)
Supplement: Supplementary file 1 — Supporting information. [file CLC-47-e24244-s001.docx]

**The effect of kidney function on guideline-directed medical therapy implementation and prognosis in heart failure with reduced ejection fraction**

**- Supplementary material -**

**Supplementary material, Table 1:** Proportion of pharmacotherapy and device therapy among the whole cohort at hospital admission, discharge, and 1 year.

| **Pharmacotherapy** | **Admission**  **(n=247)** | **Discharge (n=247)** | **1 year**  **(n=191)** |
| --- | --- | --- | --- |
| RASi, n (%) | 162 (66) | 219 (89) | 163 (85) |
| ACEI/ARB, n (%) | 143 (58) | 178 (72) | 118 (62) |
| ARNI, n (%) | 19 (8) | 41 (17) | 45 (23) |
| βB, n (%) | 169 (68) | 210 (85) | 170 (89) |
| MRA, n (%) | 144 (58) | 234 (95) | 158 (83) |
| Triple therapy (RASi + βB + MRA), n (%) | 104 (42) | 190 (77) | 139 (73) |
| SGLT2i, n (%) | 8 (3) | 24 (10) | 25 (13) |
| RASi target dose, n (%) | 53 (21) | 55 (22) | 80 (42) |
| ACEI/ARB target dose, n (%) | 45 (18) | 46 (19) | 59 (31) |
| ARNI target dose, n (%) | 8 (3) | 9 (3) | 21 (11) |
| βB target dose, n (%) | 62 (25) | 54 (22) | 69 (36) |
| MRA target dose, n (%) | 59 (24) | 169 (68) | 111 (58) |
| Triple therapy target dose, n (%) | 14 (6) | 16 (6) | 31 (16) |
| CRT-P/CRT-D, n (%) | 27 (11) | 45 (18) | 39 (20) |
| ICD, n (%) | 37 (15) | 52 (21) | 44 (23) |

ACEI: angiotensin-converting enzyme inhibitor, ARB: angiotensin receptor blocker, ARNI, angiotensin receptor neprilysin inhibitor, βB: beta-blocker, CRT-D: cardiac resynchronization therapy with defibrillator, CRT-P: cardiac resynchronization therapy pacemaker, ICD: implantable cardioverter-defibrillator, MRA: mineralocorticoid receptor antagonist, RASi: renin-angiotensin system inhibitor, SGLT2i: sodium-glucose co-transporter 2 inhibitor.

**Supplementary material, Table 2:** The main characteristics of the kidney dysfunction subgroups.

|  | eGFR (mL/min/1.73m^2^) | | | | | p |
| --- | --- | --- | --- | --- | --- | --- |
| Parameters | ≥90 | 60-89 | 45-59 | 30-44 | <30 |  |
| Male gender, n (%) | 27 (73) | 63 (79) | 34 (68) | 40 (78) | 21 (72) | 0.662 |
| Age, median [IQR], years | 47  [39-64] | 62  [51-70] | 68  [62-75] | 71  [64-78] | 72  [66-79] | <0.001 |
| Previous hospitalization primarily due to heart failure, n (%) | 10 (27) | 23 (29) | 21 (42) | 27 (53) | 17 (59) | 0.005 |
| De novo HFrEF, n (%) | 19 (51) | 50 (63) | 36 (72) | 41 (80) | 22 (76) | 0.031 |
| LVEF at admission, median [IQR], % | 23  [18-30] | 25  [20-30] | 26  [23-30] | 25  [20-32] | 25  [22-33] | 0.181 |
| Heart rate at admission, median [IQR], min^-1^ | 93  [80-110] | 92  [77-105] | 87  [79-109] | 80  [60-100] | 75  [60-86] | <0.001 |
| Systolic blood pressure at admission, median [IQR], mmHg | 121 [109-139] | 117 [102-134] | 120 [103-140] | 112 [99-129] | 115 [101-135] | 0.509 |
| Diabetes, n (%) | 9 (24) | 32 (40) | 16 (32) | 23 (45) | 20 (69) | 0.003 |
| Hypertension, n (%) | 13 (35) | 49 (61) | 31 (62) | 39 (76) | 24 (83) | <0.001 |
| Atrial fibrillation/flutter, n (%) | 10 (27) | 31 (39) | 31 (62) | 27 (53) | 15 (52) | 0.009 |
| Coronary artery disease, n (%) | 13 (35) | 30 (38) | 22 (44) | 32 (63) | 17 (59) | 0.019 |
| Creatinine, median [IQR], μmol/L | 69  [64-78] | 96  [83-106] | 116  [100-126] | 150  [138-169] | 221  [194-297] | <0.001 |
| Potassium, median [IQR], mmol/L | 4.3  [3.9-4.7] | 4.4  [4.0-4.8] | 4.4  [4.1-4.7] | 4.4  [4.1-4.7] | 4.2  [3.9-4.6] | 0.248 |

eGFR: estimated glomerular filtration rate, HFrEF: heart failure with reduced ejection fraction, IQR: interquartile range, LVEF: left ventricular ejection fraction.

**Supplementary material, Table 3A:** Independent predictors of triple therapy application at discharge.

| **Predictors of triple therapy application at discharge** | | | | |
| --- | --- | --- | --- | --- |
| **Univariate logistic regression analysis** | | | | |
|  | OR | 95% CI | | p value |
| Age (/1 year) | 0.921 | 0.893 | 0.950 | <0.001 |
| Female gender (yes) | 0.653 | 0.340 | 1.254 | 0.198 |
| Duration of hospitalization (/1 day) | 0.968 | 0.949 | 0.987 | 0.001 |
| Heart rate (/1 min^-1^) | 1.007 | 0.993 | 1.021 | 0.331 |
| Systolic blood pressure (/1 mmHg) | 1.012 | 0.998 | 1.027 | 0.099 |
| eGFR at admission (/1 mL/min/1.73 m^2^) | 1.049 | 1.032 | 1.066 | <0.001 |
| Potassium at admission > 4.5 mmol/L (yes) | 0.479 | 0.263 | 0.873 | 0.016 |
| NT-proBNP at admission (/1 pg/ml) | 0.992 | 0.988 | 0.995 | <0.001 |
| Left ventricular ejection fraction (/1%) | 1.015 | 0.972 | 1.061 | 0.497 |
| Diabetes (yes) | 0.396 | 0.217 | 0.725 | 0.003 |
| Hypertension (yes) | 0.478 | 0.245 | 0.933 | 0.030 |
| Atrial fibrillation/flutter (yes) | 0.651 | 0.359 | 1.179 | 0.157 |
| Coronary artery disease (yes) | 0.782 | 0.432 | 1.414 | 0.415 |
| CRT at admission (yes) | 0.385 | 0.167 | 0.885 | 0.025 |
| Multivariate logistic regression analysis | | | | |
|  | OR | 95% CI | | p value |
| Age (/1 year) | 0.919 | 0.881 | 0.959 | <0.001 |
| Duration of hospitalization (/1 day) | 0.974 | 0.949 | 1.001 | 0.059 |
| eGFR at admission (/1 mL/min/1.73 m^2^) | 1.016 | 0.994 | 1.038 | 0.157 |
| Potassium at admission > 4.5 mmol/L (yes) | 0.658 | 0.305 | 1.417 | 0.285 |
| NT-proBNP at admission (/1 pg/mL) | 0.994 | 0.990 | 0.999 | 0.007 |
| Diabetes (yes) | 0.371 | 0.169 | 0.818 | 0.014 |
| Hypertension (yes) | 0.998 | 0.410 | 2.427 | 0.997 |
| CRT at admission (yes) | 0.611 | 0.215 | 1.740 | 0.357 |

CI: confidence interval, CRT: cardiac resynchronization therapy, eGFR: estimated glomerular filtration rate, FUP: follow-up, HFOC: Heart Failure Outpatient Clinic, HR: hazard ratio, NT-proBNP: N-terminal pro-B-type natriuretic peptide, OR: odds ratio.

**Supplementary material, Table 3B:** Independent predictors of 1-year all-cause mortality.

| **Predictors of 1-year all-cause mortality** | | | | |
| --- | --- | --- | --- | --- |
| Univariate Cox regression analysis | | | | |
|  | HR | 95% CI | | p value |
| Age (/1 year) | 1.058 | 1.034 | 1.083 | <0.001 |
| Female gender (yes) | 1.084 | 0.600 | 1.959 | 0.789 |
| Duration of hospitalization (/1 day) | 1.014 | 0.999 | 1.029 | 0.064 |
| Heart rate (/1 min^-1^) | 0.992 | 0.980 | 1.004 | 0.215 |
| Systolic blood pressure (/1 mmHg) | 0.983 | 0.971 | 0.996 | 0.012 |
| eGFR at discharge (/1 mL/min/1.73 m^2^) | 0.975 | 0.963 | 0.987 | <0.001 |
| Potassium at discharge > 4.5 mmol/L (yes) | 1.068 | 0.632 | 1.805 | 0.807 |
| Left ventricular ejection fraction (/1%) | 0.991 | 0.953 | 1.030 | 0.648 |
| Diabetes (yes) | 1.721 | 1.019 | 2.908 | 0.042 |
| Hypertension (yes) | 1.485 | 0.832 | 2.653 | 0.181 |
| Atrial fibrillation/flutter (yes) | 1.986 | 1.162 | 3.397 | 0.012 |
| Coronary artery disease (yes) | 0.976 | 0.577 | 1.650 | 0.928 |
| CRT at discharge (yes) | 1.101 | 0.570 | 2.129 | 0.775 |
| FUP at HFOC (yes) | 0.412 | 0.228 | 0.744 | 0.003 |
| Triple therapy at discharge (yes) | 0.286 | 0.169 | 0.484 | <0.001 |
| Multivariate Cox regression analysis | | | | |
|  | HR | 95% CI | | p value |
| Age (/1 year) | 1.039 | 1.009 | 1.070 | 0.010 |
| Systolic blood pressure (/1 mmHg) | 0.983 | 0.970 | 0.997 | 0.017 |
| eGFR at discharge (/1 mL/min/1.73 m^2^) | 0.993 | 0.978 | 1.008 | 0.329 |
| Diabetes (yes) | 1.271 | 0.724 | 2.231 | 0.404 |
| Atrial fibrillation/flutter (yes) | 1.546 | 0.866 | 2.761 | 0.141 |
| FUP at HFOC (yes) | 0.501 | 0.269 | 0.933 | 0.029 |
| Triple therapy at discharge (yes) | 0.528 | 0.289 | 0.965 | 0.038 |

CI: confidence interval, CRT: cardiac resynchronization therapy, eGFR: estimated glomerular filtration rate, FUP: follow-up, HFOC: Heart Failure Outpatient Clinic, HR: hazard ratio, OR: odds ratio.

**Supplementary material, Table 4:** Comparison of neurohormonal antagonist therapy among different registries (GWTG-HF (2), SwedeHF (41), CARDIOREN registry (42)).

| Medication | Database | eGFR (mL/min/1.73m^2^) | | | | |
| --- | --- | --- | --- | --- | --- | --- |
|  |  | ≥90 | 60-89 | 45-59 | 30-44 | <30 |
| RASi (%) | GWTG-HF | 85 | 80 | 69 | 50 | 27 |
|  | SwedeHF | 96 | | 92 | 86 | 68 |
|  | CARDIOREN registry | 96 | | 95 | 85 | 68 |
|  | Current study | 100 | 100 | 77 | 86 | 41 |
| βB (%) | GWTG-HF | 90 | 89 | 88 | 86 | 80 |
|  | SwedeHF | 94 | | 93 | 92 | 92 |
|  | CARDIOREN registry | 92 | | 90 | 78 | 89 |
|  | Current study | 92 | 89 | 88 | 80 | 69 |
| MRA (%) | GWTG-HF | 45 | 40 | 35 | 26 | 14 |
|  | SwedeHF | 45 | | 44 | 37 | 24 |
|  | CARDIOREN registry | 83 | | 81 | 60 | 49 |
|  | Current study | 97 | 98 | 96 | 92 | 86 |
| Triple therapy (%) | GWTG-HF | 38 | 33 | 25 | 15 | 5 |
|  | SwedeHF | 38 | | 35 | 28 | 15 |
|  | CARDIOREN registry | 76 | | 73 | 44 | 41 |
|  | Current study | 92 | 88 | 80 | 73 | 31 |

βB: beta-blocker, eGFR: estimated glomerular filtration rate, GTWG-HF: Get With The Guidelines-Heart Failure Registry, MRA: mineralocorticoid receptor antagonist, RASi: renin-angiotensin system inhibitor.
